# Identification of differentially expressed genes from multipotent epithelia at the onset of an asexual development

**DOI:** 10.1038/srep27357

**Published:** 2016-06-06

**Authors:** Lorenzo Ricci, Ankita Chaurasia, Pascal Lapébie, Philippe Dru, Rebecca R. Helm, Richard R. Copley, Stefano Tiozzo

**Affiliations:** 1 CNRS, Sorbonne Universités, UPMC Univ Paris 06, Laboratoire de Biologie du Développement de Villefranche-sur-mer, Observatoire Océanographique, 06230, Villefranche-sur-mer, France; 2 Biology Department, Woods Hole Oceanographic Institution, Woods Hole, MA 02543, USA

## Abstract

Organisms that have evolved alternative modes of reproduction, complementary to the sexual mode, are found across metazoans. The chordate *Botryllus schlosseri* is an emerging model for asexual development studies. *Botryllus* can rebuild its entire body from a portion of adult epithelia in a continuous and stereotyped process called blastogenesis. Anatomy and ontogenies of blastogenesis are well described, however molecular signatures triggering this developmental process are entirely unknown. We isolated tissues at the site of blastogenesis onset and from the same epithelia where this process is never triggered. We linearly amplified an ultra-low amount of mRNA (<10ng) and generated three transcriptome datasets. To provide a conservative landscape of transcripts differentially expressed between blastogenic *vs.* non-blastogenic epithelia we compared three different mapping and analysis strategies with a *de novo* assembled transcriptome and partially assembled genome as references, additionally a self-mapping strategy on the dataset. A subset of differentially expressed genes were analyzed and validated by *in situ* hybridization. The comparison of different analyses allowed us to isolate stringent sets of target genes, including transcripts with potential involvement in the onset of a non-embryonic developmental pathway. The results provide a good entry point to approach regenerative event in a basal chordate.

In order to build a metazoan adult body, a universal starting point is the deployment of an embryonic and morphogenetic program inscribed in a fertilized egg. However, organisms with alternative modes of reproduction, complementary to the sexual mode, are not unusual across metazoans^1^,^2^,^3^,^4^. Species that can reproduce asexually and/or regenerate adopt ontogenetic pathways that do not rely on a fertilized egg, but that involve other cells or tissues and possibly different molecular mechanisms of development^1^,^5^,^6^. Asexual reproduction is characterized by the formation of the new organism from one part of an adult organism; this part often contains a large and heterogeneous number of cells or even complex tissues and organs^1^.

Colonial ascidians are the closest relatives of vertebrates that can adopt alternative modes of development to build an adult body besides embryogenesis^6^,^7^. During colonial ascidian embryogenesis, conserved deterministic cell specification and a stereotyped mosaic embryogenesis give rise to a tadpole larva^7^,^8^. After a short mobile planktonic phase the larva settles and metamorphoses into a sessile adult, called a zooid. From this point, a portion of epithelia situated in a spatially defined area of the body starts to bud and, in a stereotyped process called blastogenesis, gives rise to new adult zooids. Continuous blastogenic development eventually leads to the formation of a colony, where all the adult zooids share the same genotype^9^,^10^.

*Botryllus schlosseri* is one of the most well-studied species of colonial ascidians^9^,^10^,^11^. In *B*. *schlosseri,* non-embryonic development occurs mainly via a form of blastogenesis called palleal budding (Fig. 1a, reviewed in Manni *et al.* 2015^10^)^5^,^9^. While sexual reproduction, i.e. the development via embryogenesis, is seasonal and dependent on environmental conditions^12^, blastogenesis in *B. schlosseri* is a continuous, lifelong developmental process that skips all the embryonic ontogenetic and metamorphoses stages, and allows for growth and propagation of the colony. In *B. schlosseri,* the onset of the new bud relies on two ectodermal-derived mono-layered epithelia, the epidermis and the peribranchial epithelia, and possibly a population of mesoderm-derived haemoblasts (Fig. 1)^10^,^13^. Despite the fact that both the epidermis and peribranchial epithelia are ectodermal in embryonic origin^14^, these two epithelia give rise to adult functional zooids that include all putative germ layers derivatives^10^.

The coexistence of two stereotyped ontogenetic pathways within the same species, embryogenesis and blastogenesis, makes *B. schlosseri* an exceptional chordate model to study and compare different developmental programs within the same species, and even the same individual. Ultimately, allowing us to explore questions related to cell de/trans-differentiation as well as stem cell biology^6^,^11^,^15^ and ageing^16^,^17^.

While extensive literature provides detailed descriptions of morphology, anatomy and ontologies of blastogenic development (Manni *et al.*)^10^, the molecular mechanisms that trigger blastogenesis, and developmental competences in a differentiated tissue are still unknown.

In the present study we surgically isolated blastogenic tissues. Starting from an ultra-low amount of total RNA (<10 ng) we generated RNA-seq datasets from two early stages of blastogenesis and from non-blastogenic tissues. In order to obtain a conservative perspective of the genes involved in blastogenesis and to provide a reliable analysis of the transcriptomic landscapes of gene’s up-regulation in budding tissues, we compared three approaches to treat the raw data: (1) we took advantage of the recently published genome^18^ and performed differential expression (DE) using the TopHat-Cufflink pipeline^19^; (2) we also mapped our datasets to a reference transcriptome assembly, obtained from multiple developmental stages and genotypes, and proceeded to DE analyses using DEGseq package; (3) finally, we performed DE analyses using de *novo* assembling obtained after pooling only the blastogenic tissues dataset^20^,^21^. These three approaches then were compared to identify congruent list of candidate genes. Applying different strategies of assembling and mapping has provided extra-confidence on the identified stage-specific gene expression during blastogenesis.

We compiled lists of significantly over- and under- expressed genes and validated a subset by *in situ* hybridization. Our results implicate orthologous genes in conserved metazoan pathways that may be directly involved in regenerative mechanisms, and also brought to our attention a list of uncharacterized transcripts that may be specific to non-embryonic development.

## Results

### Library constructions and Illumina sequencing

**Figure 1:**
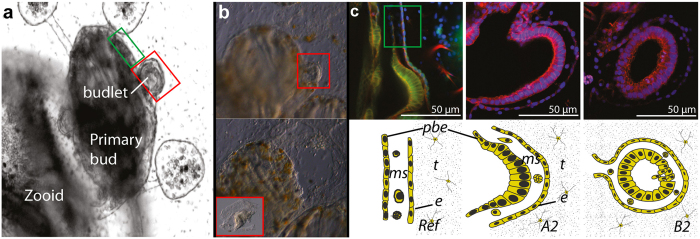
Organization of a *Botryllus schlosseri* colony and blastogenic tissues. (**a**) Part of a colony of *B. schlosseri* at stage *B2*^43^ showing the emerging budlet (red frame) and neighboring non-budding tissues (green frame). (**b**) Example of microsurgery performed to harvest the budlet, before (up) and after (bottom) ablation of the budlet. The red square in the bottom left corner shows the isolated budlet alone. (**c**) Details of the tissues sampled for the RNAseq analyses: phalloidin and dapi staining (up), and sketches (bottom). From left to right: “*Ref*” (non-budding) sample, budlet stage *A2*, and budlet stage *B2*, respectively. Sampled tissues include a monolayered peribranchial epithelia (pbe), haemoblasts included in mesenchymal space (ms), a monolayered epidermis (e), and tunic (t) with embedded cells. In the samples at stage “*A2*” and “*B2*” the two epithelia (pbe and e) acquired budding capability and present the characteristic morphology, reviewed in Manni *et al.*^10^.

To obtain the gene expression profiles at specific stages of blastogenetic development, RNA samples were isolated from three different tissues: from budlets at stage *A2* and *B2* and from non- blastogenic tissue as reference (*Ref*) (Fig. 1). For each condition a set of triplicates from three different genotypes has been originally sequenced, however, due to the inhomogeneity of one of the genotypes only duplicates have been considered in the following analyses (Supplementary Fig. S1, See Methods). The samples were sequenced on an Illumina HiSeq 2500 platform (2 × 100 bp read length), which yielded a total of ~277 million paired-end reads (Supplementary Table S1). Quality control on the raw reads was performed using FastQC.

### Transcriptome assembling and mapping approaches: reference-based and *de novo* strategies

**Figure 2:**
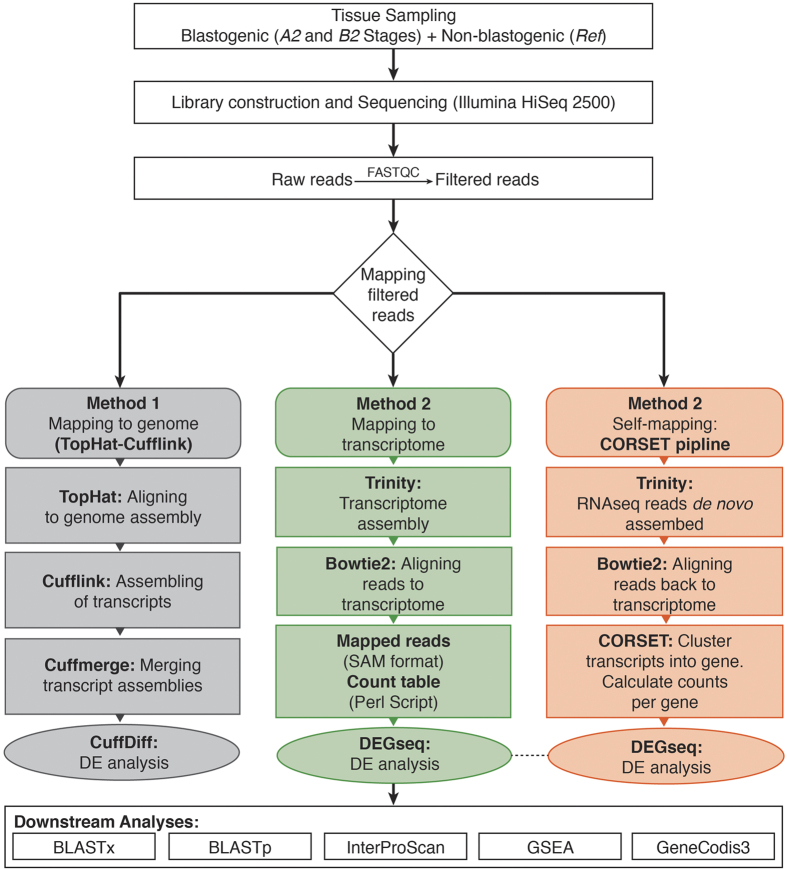
Analysis workflow A schematic overview summarizing different steps followed along with corresponding software component. Three different mapping strategies are highlighted in grey (mapping to reference genome), green (mapping to reference transcriptome) and orange (Self-mapping).

In order to provide a conservative DE analyses on the samples (see Discussion), we have applied three different approaches of sequence assembly mapping and cross-compared the differential expression data obtained from each approach (Fig. 2).

#### Reference-guided method

Starting with the straightforward approach of ‘*aligning-followed-by-assembling*’ (methodology 1), reads were first aligned to the partially sequenced reference genome assembly^18^ using TopHat2^22^ (methodology 1). This resulted in an average 46.77% (over six samples) of reads mapping (Supplementary Table S2). Mapped reads were assembled into transcripts using Cufflinks^22^, however because of a lower average mapping percentage (less than 50%) and poor DE results, we did not continue with this approach (see Discussion).

##### De-novo methods

Applying the approach of ‘*assembling-followed-by-aligning*’ (methodology 2), a *de novo* transcriptome of *B. schlosseri* has been assembled from different ESTs and RNA-seq dataset (See Methods), with a total of 373,374 assembled transcripts, averaging 357 bp in length and an N_50_ of 1458 base pairs (Table 1). Total RNA-seq reads were then mapped (separately for each sample) to the *de novo* transcriptome using Bowtie2 which resulted into 58.06% overall average alignment rate (Supplementary Table S3).

Since the reference transcriptome has been assembled from different genotypes, i.e. with high level of polymorphisms, in order to improve the mapping we adopted a third approach (methodology 3) based on a recently published pipeline CORSET^21^. Pooling the RNA-seq reads from the six samples, i.e. the duplicates from stages *A2*, *B2* and *Ref* (non-blastogenic tissue), were *de novo* assembled, using Trinity v2.0.6, yielding a total number of 230,885 transcripts with average contig length of 346 bp (Supplementary Table S3). This assembly was then used as reference for mapping. Therefore, the reads were self-mapped back to this current reference assembly, using Bowtie2, generating an average alignment rate of 79% (Supplementary Table S3).

### Conservative DE analyses of linear amplified libraries

**Figure 3:**
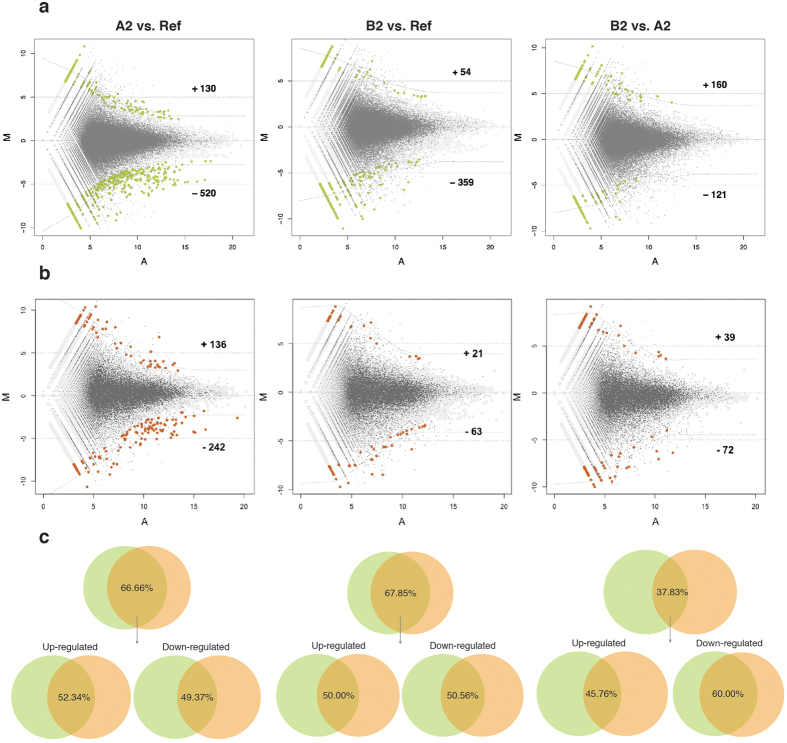
MA-plots and Venn diagrams MA-plot showing fold-change in expression generated by comparing: *A2* vs. *Ref*, *B2* vs. *Ref* and *B2* vs. *A2* (represented column wise). (**a**) Upper panel: DEGs (in green) were obtained using methodology 2; (**b**) Middle panel: DEGs (in orange) were obtained from methodology 3. Corresponding numbers of up- and down- regulated are marked with positive and negative symbols. The plots are obtained using DEGseq package, where M (Y-axis) represents the intensity ratio log_2_(fold change), and A (X-axis) represents the average intensity. (**c**) Bottom panel: Venn diagrams (top-row) showing percentage of overall DEGs identified by methodology 3 (orange circle) that overlaps with DEGs identified methodology 2 (green circle), under each three pair-wise tissue comparison. The percentage values under ‘Up-regulated’ and ‘Down-regulated’ venn diagrams (bottom-row), corresponds only to the intersection of two methodologies.

Differential expression was tested for all three pairwise comparisons between two blastogenic stages, i.e. *A2* vs. *B2*, and between blastogenic and non-blastogenic (*Ref*) epithelia: *A2* vs. *Ref*, *B2* vs. *Ref* (Fig. 1c). By using the two *de novo* methods (methodology 2 and methodology 3), differential expression was assessed using DEGseq package^23^, by applying the P-value criteria of 0.001 (default) for contigs to be statistically significant which corresponds to the z-score threshold of +/−3.3. The total number of identified DEGs along with their up-/down-regulation are summarized in Table 2 and displayed as MA-plots using different color scheme corresponding to the methodology used (Fig. 3a,b). Other package e.g. DEseq2, which is based on the negative-binomial distribution, has also been tested for DE analysis. DEseq2 has identified 1127 significant genes for *A2* vs. *Ref* that is almost double of those identified by DEGseq package, implying over-sensitivity of DEseq2 for current experimental design.

A cross comparison of differentially expressed genes (DEGs) identified under methodology 2 and methodology 3 is presented as Venn diagram illustrating the overlapped DEGs (Fig. 3c). For *A2* vs. *Ref* overall 66.66% of DEGs, identified using methodology 2, coincided with DEGs identified using methodology 3. Similarly, in the case of *B2* vs. *Ref* and *B2* vs. *A2*, an overlap of 67.85% and 37.83%, respectively, was found. The up-regulation and down-regulation contribution out of this total overlap has been separately calculated and showed with venn titled ‘up-regulated’ and ‘down-regulated’, respectively (Fig. 3, Bottom panel).

Comparing assembly statistics and differential gene expression analysis results obtained from methodology 2 and methodology 3, further downstream functional analyses were continued only using the results obtained from method 2 (See Discussion).

### Tissue specific expression of up-regulated DEGs

**Figure 4:**
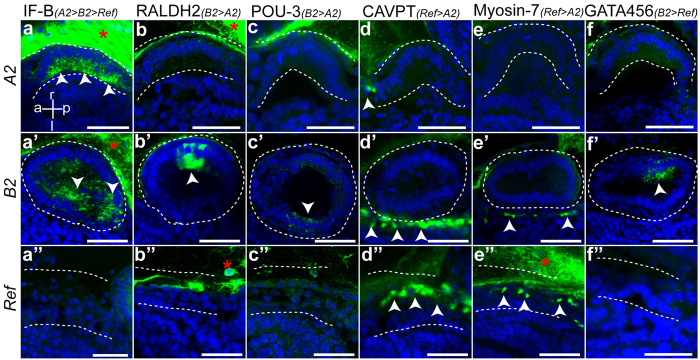
Validation of RNA-seq data revealed by FISH of DEG in dissected tissues Confocal pictures showing the expression of genes up-regulated after DEGs analysis. Green: riboprobes; blue: nuclei (DAPI). For each selected gene, FISH pictures are shown for all three collected tissues (rows of the panel). Each column shows a single gene expression pattern (gene names at the top of the columns); difference in relative expression of transcripts is indicated within parentheses. White dashed line delineates, for each tissue, the portion of collected sample for RNA sequencing. Arrowheads indicate areas of gene expression (absent when FISH experiment did not allow transcript detection). Red asterisks indicate non-specific signal due to the extracorporeal matrix, the tunic. (**a**), (a’), (b’), (c’) and (f’) shows expression in the budlet inner epithelium. (d’), (d”), (e’) and (e”) shows expression in the mantle of the primary bud (muscle fibers). All pictures are shown with the same orientation, relatively to the primary bud body axes, as represented in picture (**a**). Scale bar 40 μm.

By isolating blastogenic budlets, at stage *A2* and *B2*, and non-blastogenic tissue, we collected heterogeneous tissue samples, which included: peribranchial epithelia, mesenchymal cells (haemoblasts), epidermis and tunic with embedded tunic cells (Fig. 1c). In order to reveal the tissue specificity of over-expressed genes, but also to validate the results obtained from the DE analyses from methodology 2 (*de novo* method), we randomly selected a set of candidate genes more abundant (z-score+/−3.3) in the reference sample (*Ref*) and in blastogenic buds, and tested their expression via fluorescent *in situ* hybridization (FISH). In parallel to the selected genes, negative control with sense RNA probes were performed for each FISH experiment (Supplementary Fig. S2). FISH showed that IF-B (Intermediate filament B) mRNAs are localized in both the budlets at stage *A2* and *B2* (Fig. 4a,a’). Stage *B2* shows an expression of GATAa confined in the posterior sides of the budlet inner vesicle, in a patch of about 10 to 15 cells distally regarding the primary bud position (Fig. 4f’). At the same stage *B2* the inner vesicle shows a localized expression of RALDH2 in the same area that expresses GATAa (Fig. 4b’). Pou-3 was also differentially expressed in *B2* and weakly localized in the cells of the inner vesicle, with relatively high expression in few cells in the proximal side of the bud (Fig. 4c’). The reference samples confirmed the overexpression of CAVP-target-PL and Myosin-7 in muscle cells wrapping the primary bud and located between the peribranchial epithelium and the epidermis (Fig. 4d”,e”). They were also detected, lining underneath the budlet in *B2* (Fig. 4d’,e’), and sometimes flanking the budlet in *A2* (Fig. 4a). Additionally, FISH experiments revealed that several significant DEGs were expressed in bud-adjacent tissues, e.g. blood cells or germ cells (Supplementary Fig. S3).

### Functional annotation and classification

To gain biological insights into the identified DEGs identified under methodology 2 (*de novo* method), functional annotations were retrieved using the following programs: BLASTx, BLASTp, InterProScan and enrichment analysis were preformed using Genecodis3 and GSEA (see Methods). All the results obtained from functional and enrichment studies, on the differentially expressed genes, under each pairwise tissue comparisons, were integrated in Supplementary Data S1, S2, S3, and S4. Performing BLASTx searches on the DEGs against NCBI non-redundant (nr) database resulted into 432, 263 and 151 transcripts with known genes under *A2* vs. *Ref*, *B2* vs. *Ref* and *B2* vs. *A2*, respectively. Further categorizing of top hits of BLASTx, in order to understand the species distribution, first two top-hit species were *Ciona intestinalis* and *Branchiostoma floridae and* only 0.41% (only 15 hits) were from of *Botryllus schlosseri* published sequences (Supplementary Fig. S4b). Domain level searches were performed against InterProScan database 5RC6, choosing default analyses e.g. Pfam, SMART, PRINTS, ProSiteProfiles, ProSitePattern and -goterm flag to generate GO term mapping. On average (over all three pairwise tissue comparisons) 48% of the DEGs have been classified with different InterPro signatures.

### Functional enrichment analyses

For the interpretation of biological roles of identified DEGs, functional enrichment analysis was implemented using a web-based tool Genecodis3 by providing list of human homologs of DEGs identified under methodology 2 (*de novo* method) as an input (Table 2). Given this query sets, Genecodis3 was used to provide the enrichment for biological annotations namely: Gene Ontology (GO) categories, InterPro, Transcription Factor and KEGG pathways, using following statistical parameters: (i) In case of modular enrichment analyses for the combination of annotation to appear a minimum support from 3 genes was required, (ii) for P-value calculation hypergeometric statistical test was selected (significance threshold P-value < 0.05), (iii) for multiple hypothesis testing FDR estimation was utilized for P-value correction. Statistical relevance for each category are reported in Supplementary Data S2, S3, and S4 and graphical representations of the enriched annotations are reported in Supplementary Figs S5–7. Specifically, comparing the annotations for differentially expressed genes between two blastogenic stages *B2* vs. *A2* revealed significant overexpression of the following genes at *B2* stage: RDH13 (Retinol dehydrogenase 13), ALDH1A2 (Aldehyde dehydrogenase) characterized under GO:0016491 MF oxyreductase activity with FDR of 0.046 (using SEA, see Methods) and FDR = 0.022 (using MEA, see Methods). InterPro annotation reported the genes containing the POU3F4 domain (IPR016362, FDR = 0.0097). While examining *B2* vs. *Ref*, GATA4/5/6 (IPR016357 and IPR00619) was found to be significantly enriched in the *B2* stage with an FDR of 0.01. Further inspection of up-regulated gene sets identified Vimentin (IF-B) at stage *A2* and Myosin (Myosin-7) in *Ref* tissue. Pie charts and tag clouds are summarized under Supplementary Figs S5–7. Further applying the gene set enrichment analysis (using GSEA, See Methods) although reproduced similar results in terms of interested candidate genes, but the enriched gene sets showed higher FDR value (FDR = 1), limited the confidence on the resulted enriched sets using GSEA.

## Discussion

Non-embryonic developmental pathways are recurrent among metazoans and are often associated with high regenerative capabilities^6^,^24^,^25^,^26^. In the case of asexual propagations, for example through budding, part of an adult body re-starts a developmental program that gives rise to the clonal new adult^1^,^5^. In this study for the first time we isolated budding tissues from a chordate, the colonial ascidian *B. schlosseri*, revealing their transcriptomic landscapes. In addition we highlighted the differences in terms of gene expression between budding tissues and adult tissues without budding capabilities, but with supposedly similar ontogenetic origin, i.e. derived from the same embryonic germ layer^10^.

In colonial ascidians such as *B. schlosseri*, a single colony can be divided (sub-cloned) into different parts and grow separately, allowing sets of tissues from isogenic colonies to be obtained. In this manner, we were be able to isolate two spatially contiguous set of tissues, i.e. budding and non-budding, but also two temporally consecutive budding stages (*A2* and *B2*) of the same genotype while avoiding the risk of altering the transcriptomic profile with prolonged surgical procedures. On the other hand, since the stages of budding are highly stereotyped, we decided to choose different genotypes for each of the replicates. The choice of biological instead of technical replicates produced a higher variability during the DE analyses (Supplementary Fig. 1)^27^, leading to the exclusion of one of the original genotypes. In order to cope with such variability, and to avoid potential biases introduced by the stem of linear amplification we adopted multiple approaches to produce the DE data. The differential expression analysis using Cuffdiff^22^, identified only 75 transcripts with significant differential expression (FDR < 0.05) in case of *A2* vs. *Ref*, while for the other pairwise comparisons none of the transcripts were found to be statistically significantly differentially expressed. Functionally exploring the significant 75, by using BLASTx against non-redundant database, revealed high homology with the solitary ascidian *Ciona intestinalis* (proteins dominated with 56%) and only 2% of blast hits matched to published *B. schlosseri* sequences (Supplementary Fig. S4a). Since such an approach relies on a robust splice based aligner for genome guided transcriptome reconstruction, its efficacy is integrally limited by the quality of *Botryllus* genome, which in the current assembly state is not satisfactory. For this reason the other two methodologies based on *de novo* transcriptomes were adopted. As methodology 3 (applying CORSET clustering algorithm) showed a higher mapping percentage (Supplementary Table S3), so at first it appeared as a better approach to further analyze the sequencing data. Though on one hand, an additional advantage of methodology 3 was to avoid polymorphism issue with a transcriptome resulted from a more diverse biological data (methodology 2) that could affect mapping percentage. While on the other hand, a fairly common concern with *de novo* approaches involves incomplete assembling which might have consequences on the statistical analysis. Two main parameters that can influence estimation of expression and subsequent differential analysis are: (i) the numbers of contigs per gene (ii) and their sequence length. Major aspects contributing towards the elevated number of contigs include: a biological aspect where Trinity assembler treats different splice forms as different contigs, and a technical aspect which is linked to the insufficient overlap and coverage, leading to production of fragmented transcripts. Further, comparing the sequence length of assemblies resulted from methodology 2 and methodology 3, showed a higher average contig length value for methodology 2 (Table 1). These results were expected as the reference transcriptome used in methodology 2 comes from diverse source, on contrary to methodology 3 that include reads only from this study.

Fragmented transcripts also have statistical consequences on the differential expression analysis. Each smaller contig will contribute towards a weaker average number of count (A, x-axis of MA-plot) and this average number of counts have a direct impact on statistical significance of differential expression, e.g. for a same value of log_2_ (fold change) (M, y-axis of MA-plot), genes with a strong expression values (A, x-axis of MA-plot) would statistically be more significant than the weakly expressed genes. To ensure that methodology 2 provided better statistical values, we compared the Z-scores for genes overlapping between methodology 2 and methodology 3 and plotted the ratio of two Z-scores using boxplot (Supplementary Fig. S8). As expected, the ratio is greater than one for most of the genes, under all pairwise tissue comparisons, implying the greater Z-scores from methodology 2 than from methodology 3, which in turn suggested a reason for higher differences in the resulted number of differentially expressed genes between two methodologies (Table 2).

The functional annotations provided here are based on the human gene ontology. Still they allowed the identification of transcripts involved in stem cell behavior which are up-regulated in the blastogenic epithelia. For instance, transcription factors pleiotropically linked to stem cell migration (e.g. KITLG), telomere maintenance (e.g. POT1) and also tumor suppressors (e.g. TSC2). While further targeted data mining is required, these molecular clues suggest the involvement of a stem cell based process in the early steps of asexual development^5^,^6^,^28^. In addition, the DE analyzed between two budding stages highlighted the presence of transcription factors notably involved in embryonic developmental processes (e.g. GATA, Pitx, Tbx or RALDH. Supplementary Data S1), supporting the co-option of embryonic regulatory networks during asexual and regenerative developments, as has already been suggested in previous candidate gene approaches^29^,^30^,^31^,^32^. The activation of BMP, MAPK and Retinoic acid pathways also confirm the re-use of conserved signaling pathways during asexual development. The discovery of conserved developmental genes involved in budding has a clear potential to be tested for conservation across other metazoan taxa, including distantly related species of marine invertebrates, e.g. sponges and cnidarians. The up-regulation of around 10% of uncharacterized proteins in budding epithelia, potentially involved in the onset of asexual development processes, may suggest the presence of taxa specific proteins, which could provide insight in the evolution of adult pluripotent epithelia. The patterns of expression of these genes and pathways can be followed during the blastogenetic process via *in situ* hybridization^29^,^33^, and their function dissected via siRNA, as previously reported in “gene-by-gene” approaches^34^,^35^,^36^,^37^.

The datasets here provided are the first tissue specific transcriptomes of asexual development in a chordate, and represent the initial step to characterize, on a large scale, conserved and novel molecular players acting on an epithelium with pluripotent characteristics. The approach adopted, which couples ultra-low input RNA with stringent data analyses, represents a proof of principle that can be successfully applied in further steps, i.e. the collection of additional blastogenic stages, and different reference tissues, not only in *B. schlosseri* but also in other ascidians and budding metazoans. Whereas vertebrates have limited regenerative capacity, many invertebrate models such as colonial ascidians can rebuild their bodies completely in response to injury^38^,^39^ or as part of their life cycle^5^,^40^,^41^. Unlike mammals, where genomic and transcriptomic data are abundant, these models are often poorly annotated due to lack of sequence data^42^. The three transcriptomes, together with their DE analyses, provide a tissue specific toolset for the study of the regeneration of complex structures. In order to provide further validation these datasets, more expression (via FISH) and functional testing (i.e. via siRNA) are still required. However, these tools offer the captivating perspective of performing insightful comparisons with vertebrate regenerative processes, but it also can be of potential use in comparative studies apt to address the evolution of coloniality and complex life cycle^11^,^26^.

## Methods

### Tissue sampling and RNA isolation

The *Botryllus schlosseri* used for experiments were collected from Villefranche-sur-mer (43°42′18″N 7°18′45″E), raised on glass slides in a marine-culture system as described in Langenbacher *et al.*^33^. Blastogenetic development was staged according to the Lauzon staging method^43^. Three genetically distinct and isogenic colonies, labeled AH, AS and AX, respectively, were isolated on glass slides and sub-cloned, i.e. divided into different parts that can grow separately (Fig. S1). To analyze the onset of blastogenesis, a microsurgery technique was used to isolate the regenerative tissues from budlets (stage *A2* and *B2*) and from non- blastogenic epithelia as Reference (Fig. 1). Harvested tissues were directly stored in lysis buffer and PolyA mRNA was extracted with Magnetic mRNA Isolation Kit (New England Biolabs).

### RNA-seq library preparation and Illumina sequencing

PolyA mRNA samples were sent to SeqWright Genomic Services (GE Healthcare, Houston), where the libraries were constructed and linearly amplified using ClonTech SMART Sample Prep (ClonTech). Illumina HiSeq 2500 sequencing was performed and a dataset of approximately PE100 70M RNA-seq reads were obtained for each stage and for each genotype (biological triplicates). The overall read quality was checked using FastQC (“Andrews, S., FastQC: A quality control tool for high throughput sequence data, http://www.bioinformatics.bbsrc.ac.uk/projects/fastqc/, 15/09/2014”).

### Transcriptome assembly and alignment strategies

Due to its high variance, the sample AX was excluded from further analyses (Supplementary Fig. S1) and the overall workflow is summarized schematically in Fig. 2.

### Mapping to Genome (Methodology 1)

A total of approximately 277 million paired-end reads were first mapped to the reference genome (580 Mbp) of *B. schlosseri* [European Nucleotide Archive accession number HF548551 (“Voskoboynik, A., *Botryllus schlosseri complete mitochondrial genome, isolate sc6a-b*, http://www.ncbi.nlm.nih.gov/nuccore/HF548551, 22/09/2014) using TopHat v2.0.11-Cufflinks v2.1.1 pipeline. Bowtie2 (v2.1.0) was used for indexing the reference genome and subsequently aligned reads were assembled into transcripts using the Cufflinks package. After assembling all samples separately, assemblies were merged using Cuffmerge. Cufflinks measures the abundance of transcripts as FPKM. Finally, differential gene expression was tested using Cuffdiff package based on following criterions: FDR (q-value) <0.05, and fold change >2.

### Reference transcriptome assembly and mapping (Methodology 2)

A reference transcriptome of *B. schlosseri,* has already been assembled, using the Trinity^44^ pipeline, by combining published ESTs databases^45^ and a transcriptomic dataset^46^ (Table 1). This transcriptome assembly is available at Octopus Bioinformatics Server (“Tiozzo, S., http://octopus.obs-vlfr.fr/public/botryllus/blast_botryllus.php, 07/01/2015”) for public use. Reads were mapped to this reference transcriptome by using Bowtie2 (v2.1.0) with the number of mismatches allowed (-N) set to 1 and -a option to report all alignments. The read count table was prepared using an in-house Perl script. Changes in gene expression were identified using DEGseq package^23^. For significant differences in expression, P-value and FDR (adjusted P-value or Q-value) thresholds were set to 0.001. The choice of using DEGseq package for this study relies on evaluation checks performed by comparing its results with results obtained using other packages that are based on negative binomial distribution (e.g. DEseq2, edgeR).

#### Self-mapping (Methodology 3)

High numbers of contigs produced per gene by *de novo* transcriptome assembly make differential expression analysis challenging. A recently published CORSET pipeline^21^, improves the statistical power of differential expression by clustering contigs to gene. The pipeline involves following three steps: (i) *de novo* assembly: all the reads were pooled keeping the paired end information intact and then were assembled using Trinity v2.0.6. (ii) Self-mapping: reads were then mapped back to the current assembled transcriptome using Bowtie2. Samtools (v.1.2) was used to produce the alignment in bam format^47^. (iii) Clustering and abundance estimation: CORSET groups the transcripts hierarchically into genes and calculates expression levels for each cluster (counts per gene). This algorithm applies a “bottom up” approach of hierarchical clustering of transcripts into gene based on two criterions: (i) distance between any two contigs depends on the number of reads shared between them (ii) clustering of these two contigs will be confirmed if and only if a constant relative expression level is maintained under two test conditions. After clustering transcripts, statistical tests for differential expression were performed on the resulting gene level count (number of reads overlapping a gene) data using the DEGseq package^23^, with P-value threshold of 0.001.

Conclusively, a cross comparison was made over the differentially expressed genes (DEGs) identified under two different methodologies (methodology 2 and methodology 3) of sequence mapping and was shown using venn representation. All the subsequent functional annotations were carried out on the dataset of DEGs obtained using methodology 2.

### Functional annotation and gene enrichment analysis

In order to find similarity with known genes and to predict their functions, multiple functional analyses on the differentially expressed contigs were performed. Sequence comparisons were performed using BLASTx and BLASTp against the NCBI non-redundant (nr) protein database (“ ftp://ftp.ncbi.nlm.nih.gov/blast/db/FASTA/nr.gz, 18/04/2015). All homology searches against NCBI non-redundant (nr) database were carried out on an HTC cluster facility provided by ABiMS, Station Biologique de Roscoff (http://abims.sb-roscoff.fr/resources/cluster, 18/04/2015).

Functional domain searches were conducted using InterProScan^48^ incorporating protein signature from Pfam, SMART, PRINTS, PROSITE, Gene3D, SUPERFAMILY and PANTHER. Single peptide cleavage location and transmembrane helices were predicted using SignalP and TMHMM, respectively.

For functional enrichment analysis GeneCodis3^49^ (“Tabas-Madrid, D., *Gene Annotation co-occurrence discovery*, http://genecodis.cnb.csic.es, 11/05/2015) was used, which algorithmically incorporates two analyses: (i) Singular Enrichment Analysis (SEA) where the statistics are calculated taking single annotation term one at each time; (ii) Modular Enrichment Analysis (MEA) fundamentally acquires the SEA background additionally and produces an inter-relationships among the annotation reference terms (‘term-term relationship’) thereby providing a global picture at the biological network level. For enrichment analyses human homologs of differentially expressed *Botryllus* genes were provided (see methodology 2) and a hypergeometeric test was selected as the statistical parameter to calculate the P-value and FDR for enrichment. The extracted annotations involve: GO terms (Biological processes, Molecular Function, Cellular Component)^50^, InterPro^51^ motif and Transcription Factors^52^ from MSigDB. Further gene set enrichment analysis was also performed using GSEA (**G**ene **S**et **E**nrichment **A**nalysis) software v2.2.0 (“Gene Set Enrichment Analysis, www.broadinstitute.org/gsea/index.jsp, 15/06/2015) separately against two a priori defined gene sets collections: C2 (c2.all.v5.0.symbols), which is a collection of 4725 gene sets from various online pathways (http://www.broadinstitute.org/gsea/msigdb/collection_details.jsp#C2,15/06/2015) and C5 (c5.all.v4.0.symbols) includes 1454 gene sets based on GO annotations (http://www.broadinstitute.org/gsea/msigdb/collection_details.jsp#C5, 15/06/2015) Other settings for the GSEA run include: (i) collapse dataset to gene symbols = TRUE and (ii) number of gene set permutations were set to 1,000.

#### Data access

Transcriptome assembly is available at the Octopus database (BioInformatique, http://octopus.obs-vlfr.fr/public/botryllus/blast_botryllus.php, 04/01/2016) hosted by CNRS/UMR7009 and sequences for differentially expressed genes can be retrieved from Octopus: BioDev Bioinformatics Server, http://octopus.obs-vlfr.fr/public/botryllus/fastacmd_riccipaper.html, 28/04/2016) using contig identifier (see Supplementary Data S1) under the sequence identifier section.

### Fluorescent *in situ* hybridization

For each gene, couples of forward and reverse primers were designed in order to obtain amplified fragments between 500 and 1200 bp. A T7 promoter sequence was added at the 5′ end of each reverse primer. Fragments of interest were amplified with the Phusion^®^ High-Fidelity DNA Polymerase (NEB, M0530S) using the forward and modified reverse primers. PCR product was used for sequencing and for RNA probe synthesis (1 μg) with Digoxygenin-UTP labeling and T7-RNA Polymerase kit (Life Technologies, 18033-019). FISH was conducted as previously described^35^ avoiding proteinase K treatment and extending both hybridization and antibody incubation time to 72 h. Counterstaining was performed with Hoechst 33342 (10 μg/ml in PBS) before mounting in glycerol and imaging with a confocal Leica TCS SP5 microscope. Primer sequences used for FISH experiments can be found in Supplementary Table S4.

## Additional Information

**How to cite this article**: Ricci, L. *et al.* Identification of differentially expressed genes from multipotent epithelia at the onset of an asexual development. *Sci. Rep.* **6**, 27357; doi: 10.1038/srep27357 (2016).

## Supplementary Material

Supplementary Information

Supplementary Data 1

Supplementary Data 2

Supplementary Data 3

Supplementary Data 4

## Figures and Tables

**Table 1:** Summary statistics of transcriptome assemblies obtained using Trinity (v2.0.6) under methodology 2 and 3.

Statistics	Method 2:Reference transcriptome assembly	Method 3:CORSET pipeline
Total trinity transcripts	373,374	230,885
Total trinity genes	210,144	160,478
Total assembled bases (bp)	284,227,628	138,007,268
Median contig length	357	346
Average Contig	761.24	597.73
N25 (bp)	2,736	1,888
N50 (bp)	1,458	857
N75 (bp)	528	383
Total GC count (bp)	125,359,234	59,973,722
GC content (%)	44.11	43.46

**Table 2:** Number of DEGs and corresponding number of human homologs, for each pairwise tissue comparison, identified under methodology 2 and 3.

Samples	Method 2. Reference Transcriptome			Method 3. CORSET pipeline	
	DEGs (#)	up/down	Human homologs (#)	DEGs (#)	up/down
*A2* vs. *Ref*	650	130	523	378	136
		520			242
*B2* vs. *Ref*	413	54	297	84	21
		359			63
*B2* vs. *A2*	281	160	197	111	45
		121			66
