# Supplementary material for: Identification of differentially expressed genes from multipotent epithelia at the onset of an asexual development
Source: Sci Rep. 2016 Jun 6;6:27357. doi: 10.1038/srep27357 (PMC4893630; doi:10.1038/srep27357)
Supplement: Supplementary Information [file srep27357-s1.pdf]

Supplementary Tables and Figures

Identification of differentially expressed genes from multipotent epithelia at the onset of an asexual development

Lorenzo Ricci[1*], Ankita Chaurasia[1§*], Pascal Lapébie[1], Philippe Dru[1], Rebecca R. Helm[2], Richard R. Copley[1], Stefano Tiozzo[1]

[1] CNRS, Sorbonne Universités, UPMC Univ Paris 06, Laboratoire de Biologie du Développement de Villefranche-sur-mer, Observatoire Océanographique, 06230, Villefranche-sur-mer, France;

[2] Biology Department, Woods Hole Oceanographic Institution, Woods Hole, MA 02543, USA

[§]Corresponding author

*Equal contribution

Sample	RNA Conc.	Sequencing Yield (Mb)	Raw Reads (#)
AH_A2	< 10 ng	13,098	52,593,299
AH_B2		8,974	36,034,156
AH_ref		12,855	51,619,781
AS_A2		14,458	58,049,211
AS_B2		10,865	43,622,646
AS_ref		8,853	35,545,564
**Total**			**277,464,657**

Supplementary Table S1: Summary of sample preparation and sequencing for RNA-seq in *B.schlosseri*.

TopHat	Input reads		Mapped to Genome				
**Samples**	**Left**	**Right**	**Left**	**Right**	**Overall Align %**	**Aligned pairs**	**Concordant pair alignment rate**
**AH_A2**	52,593,299	52,593,299	24,730,401 (47.1% of input)	24,107,206 (45.8% of input)	46.40%	18,598,073	31.30%
**AH_B2**	36,034,156	36,034,156	17,590,292 (48.8% of input)	17,113,730 (47.5% of input)	48.20%	13,226,320	32.20%
**AH_ref**	51,619,781	51,619,781	24,384,113 (47.2% of input)	23,773,211 (46.1% of input)	46.60%	18,434,710	32.10%
**AS_A2**	58,049,211	58,049,211	27,869,581 (48.0% of input)	27,211,909 (46.9% of input)	47.40%	20,889,647	32.00%
**AS_B2**	43,622,646	43,622,646	19,999,057 (45.8% of input)	19,450,376 (44.6% of input)	45.20%	14,900,593	30.30%
**AS_ref**	35,545,564	35,545,564	16,878,044 (47.5% of input)	16,367,528 (46.0% of input)	46.80%	12,491,965	31.50%
**Average**					**46.77%**		**31.57%**

Supplementary Table S2: Statistics of alignment of RNA-seq reads to the *B. schlosseri* genome using TopHat mapper.

Sample	Mapping to Genome (TopHat)	Mapping to transcriptome	Self-mapping CORSET
AH_A2	46.40%	57.99%	78.51%
AH_B2	48.20%	59.17%	79.58%
AH_ref	46.60%	57.84%	80.78%
AS_A2	47.40%	58.59%	79.48%
AS_B2	45.20%	57.09%	78.24%
AS_ref	46.80%	57.66%	77.55%
**Average**	**46.77%**	**58.06%**	**79.02%**

Supplementary Table S3: Sample-wise mapping percentage obtained under three different methodologies, using Bowtie2 (v2.1.0).

Gene	Contig accession number	Developmental Stages	Z-score	Primer sequence	
CAVPT	comp559506_c1_seq2	*A2* vs. *Ref**	-6.538108242	forward	TATCGGTGATACAAGGCGCA
				reverse	TGTGATCCTGTCCCATCTCG
GATA456	comp560039_c4_seq1	*B2** vs. *Ref*	4.357227969	forward	GTCATCTGTCGCTGTGTGCT
				reverse	CCGTACATCGGTGAGGAGTT
POU3	comp523660_c2_seq1	*B2** vs. *A2*	3.50349629	forward	ATAGTGAATTCGGTCTGCGC
				reverse	AGAGGCCGTAACTTGCACAT
IF-B	comp555930_c0_seq2	*A2** vs. *B2*	4.281588398	forward	CAACGCTGACACAAGAGCTT
		*B2** vs. *Ref*	3.553348996	reverse	TCGGGAGCAGAATCGAGTAC
RALDH2	comp563791_c4_seq5	*B2** vs. *A2*	4.58621399	forward	AACAAATCACCGGGTCTTGC
				reverse	TGCGTTGTCCACCTCTGTAT
Myosin7	comp566480_c1_seq1	*A2* vs. *Ref**	-6.700399441	forward	TCGAAGTCCAAGCAATCCCT
				reverse	GCGCCTCGTACTTCTTCTTG

Supplementary Table S4: Summarizing genes selected for validation, developmental tissue under comparison, (*) symbolizes up-regulation, Z-scores and primer sequences for FISH.

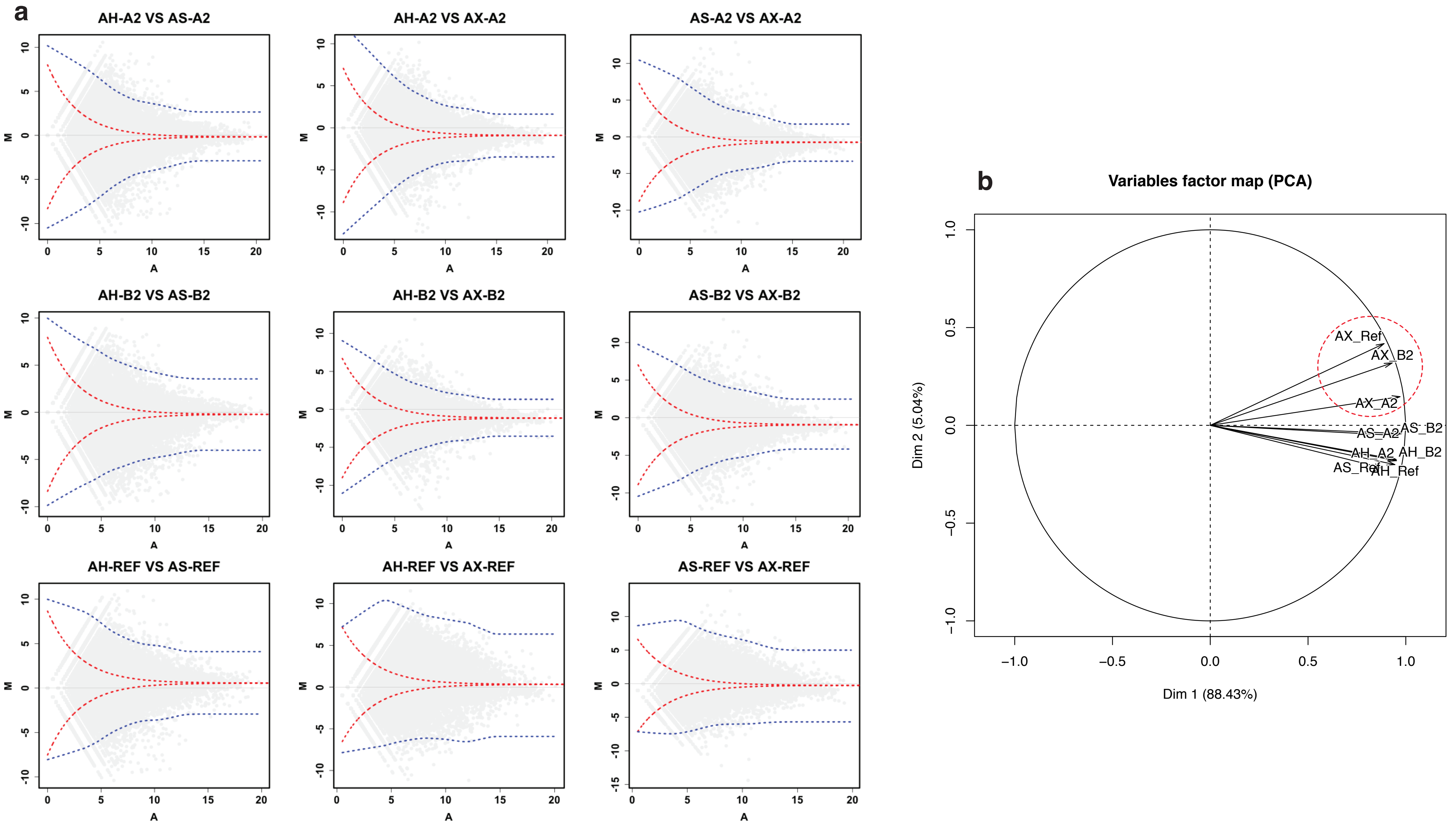

	AH vs. AS	*AH vs. AX*	*AS vs. AX*
A2	*A2*(AH vs.AS)	*A2*(AH vs.AX)	*A2*(*AS* vs.*AX*)
B2	*B2*(*AH* vs.*AS*)	*B2*(*AH* vs.*AX*)	*B2*(*AS* vs.*AX*)
Ref	*Ref*(*AH* vs.*AS*)	*Ref*(*AH* vs.*AX*)	*Ref*(*AS* vs.*AX*)

Supplementary Fig. S1: Sample variation and correlation circle plot

(a) MA-plots generated using CTR method, under DEGseq package. For all three samples *A2*, *B2* and *Ref* (represented row-wise), CTR method checks whether the variation between a pair of replicates is explained using random sampling. Red and blue dotted lines corresponds to the 'theoretical' and 'estimated' 4-fold standard deviation, respectively. Table underneath instructs the particular pair of replicates (represented column-wise) tested under each sample and corresponds to MA-plots shown above. (b) Correlation circle plot showing the projection of variables (9 samples) used for principal component analysis. The first two components explains 93.47% of total variation.

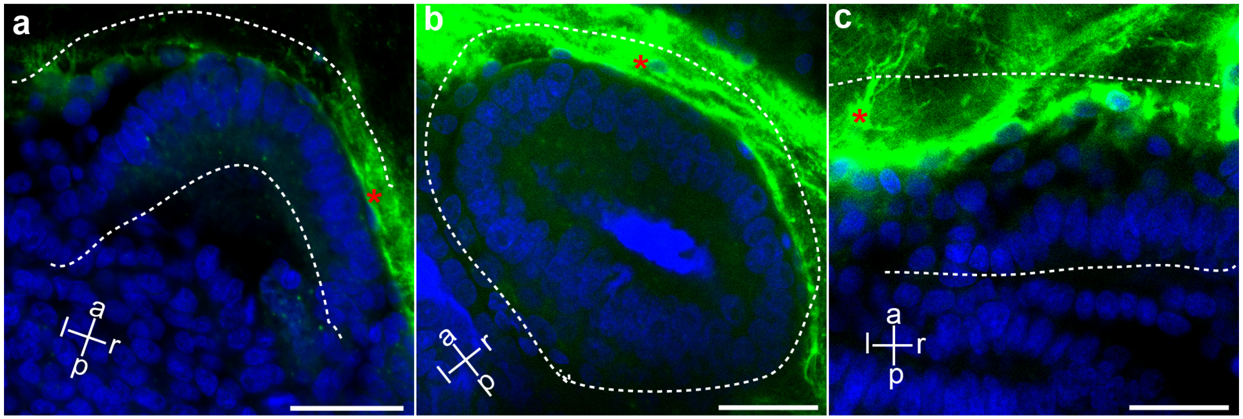

Supplementary Fig. S2: Negative control for FISH experiment
Confocal pictures of the tissues collected for RNAseq experiments. In green: non-specific signal. Nuclei are counterstained with Hoescht. Note that the variable intensity of non-specific signal, mostly due to the presence of a polysaccharidic matrix embedding the whole animal, the tunic (marked with a red asterisk). (a) and (b), palleal budlets in *A2* and *B2*, respectively; (c) Reference tissue, i.e. non-budding region of the peribranchial epithelium. Samples were hybridized with sense RNA probes, designed on *Botryllus* gene sequences, such as Piwi. Axes indicate primary bud orientation (located at the bottom of the picture). Scale bar: 25µm approximately.

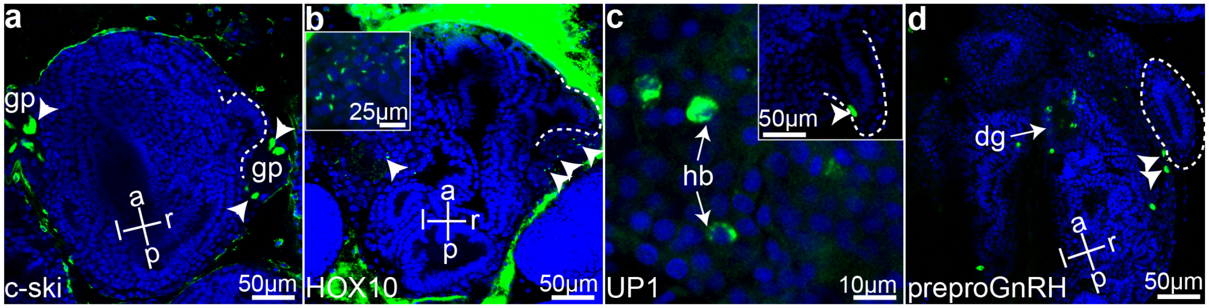

Supplementary Fig. S3: Gene expression in non-budding tissues

Confocal pictures showing expression of four differentially expressed transcripts suggesting tissue contamination. Green: riboprobes; blue: nuclei. From (a) to (c) colony at stage *A2*; (d) colony at stage *B2*. White dashed lines delineate the budlet. Arrowhead show cell expressing a target gene located in, or at immediate proximity of the budlet. Name of the genes are indicated at the bottom left corner. (a) and (b) show cells expressing the target genes were found mainly in gonad tissue, located under the budlet inner epithelium, and adhering to it. (c) shows expression of Uncharacterized Protein 1 in blood cells. Note: UP1 is a *Botryllus* gene showing no conserved domain in its predicted protein sequence. (d) shows expression of pre-pro-gonadotropin releasing hormone gene by neurons emanating from the central nervous system and progressively wrapping the primary bud. Framed pictures in (b) and (c): details of HOX10 and UP1 in gonad tissue and between the budlet and its epidermis, respectively. Legends: gp: gonad primordium; hb: heamoblast; dg: dorsal ganglion.

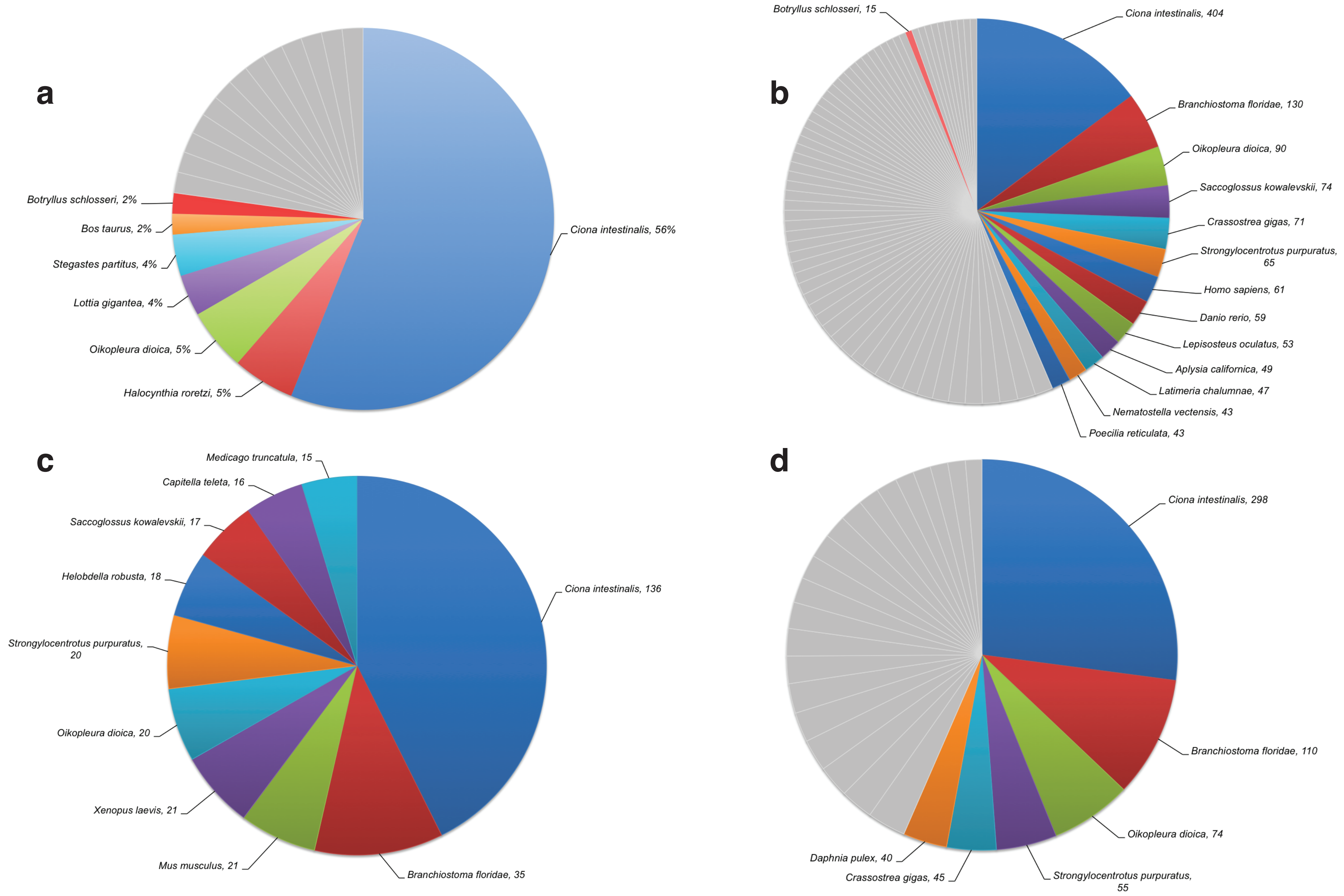

Supplementary Fig. S4: BLASTx species distribution

Species distribution of BLASTx top-hits obtained querying identified differentially expressed genes against the non-redundant (nr) database. (a) For pair *A2* vs. *Ref* significant-DEGs obtained after mapping reads to the reference genome (methodology 1). BLASTx top-hits have been labeled with corresponding percentage. (b), (c), (d) Significant-DEGs obtained after mapping to reference transcriptome assembly (methodology 2): (b) *A2* vs. *Ref*, (c) *B2* vs. *A2* and (d) *B2* vs. *Ref*. Pie-charts b, c, d only includes species with frequency ≥ 15. Pie Charts a, b, d only include prominent species shown with color coding and the rest in grey to ensure legibility; only *B.schlosseri* has been marked in particular to highlight its low frequency of occurrence.

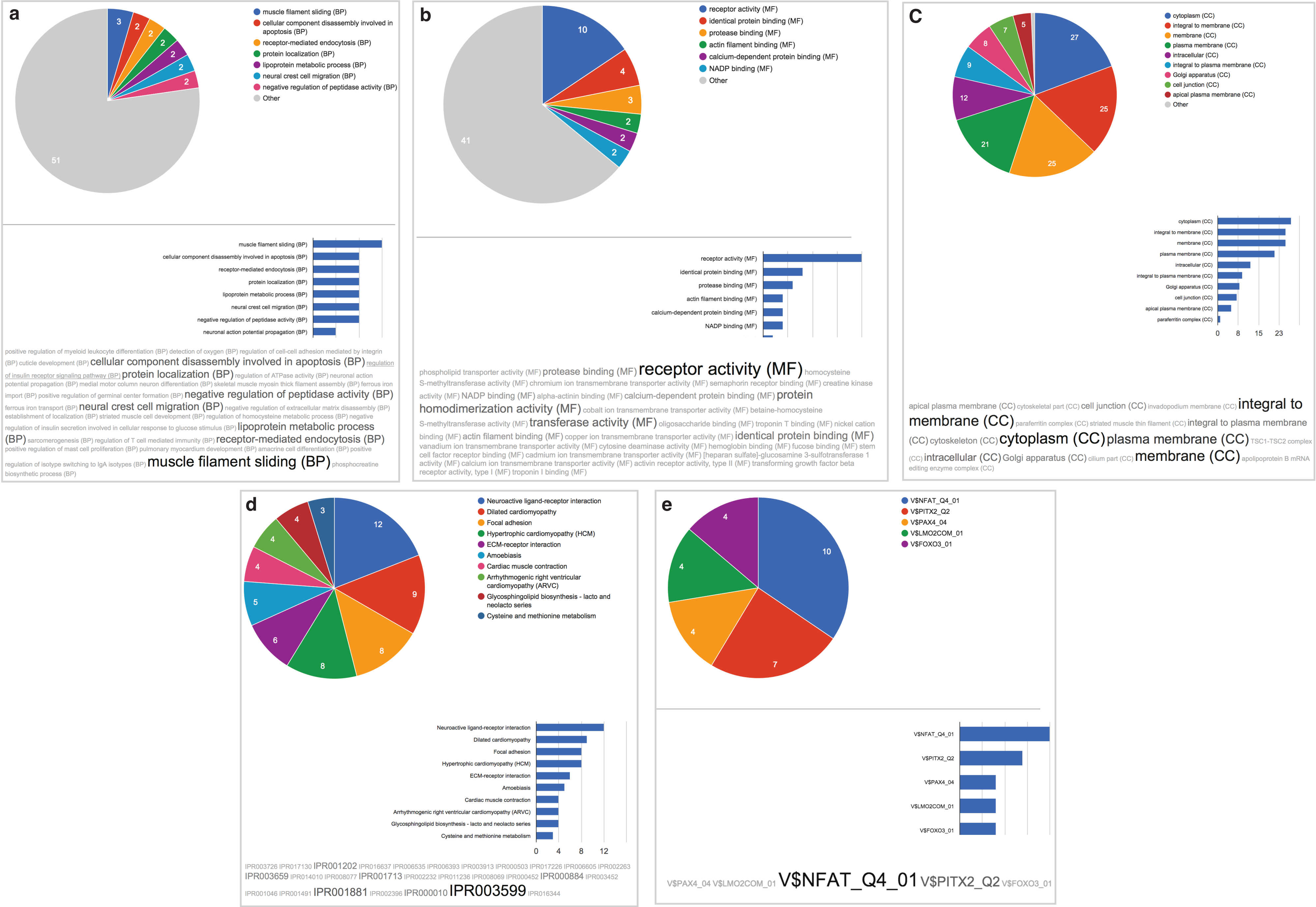

Supplementary Fig. S5: Enrichment pie charts in *A2* vs. *Ref*

Snap shots of the interactive pie charts and bar graphs obtained from Gencodis3, under following enriched annotations: (a) GO Biological processes; (b) GO Molecular function; (c) GO cellular component; (d) InterPro motifs; and (e) Transcription factors, for up-regulated DEGs identified between *A2* vs. *Ref*. Size of the slices and the length of the bars are proportional to the number of genes corresponding to the assigned annotation.

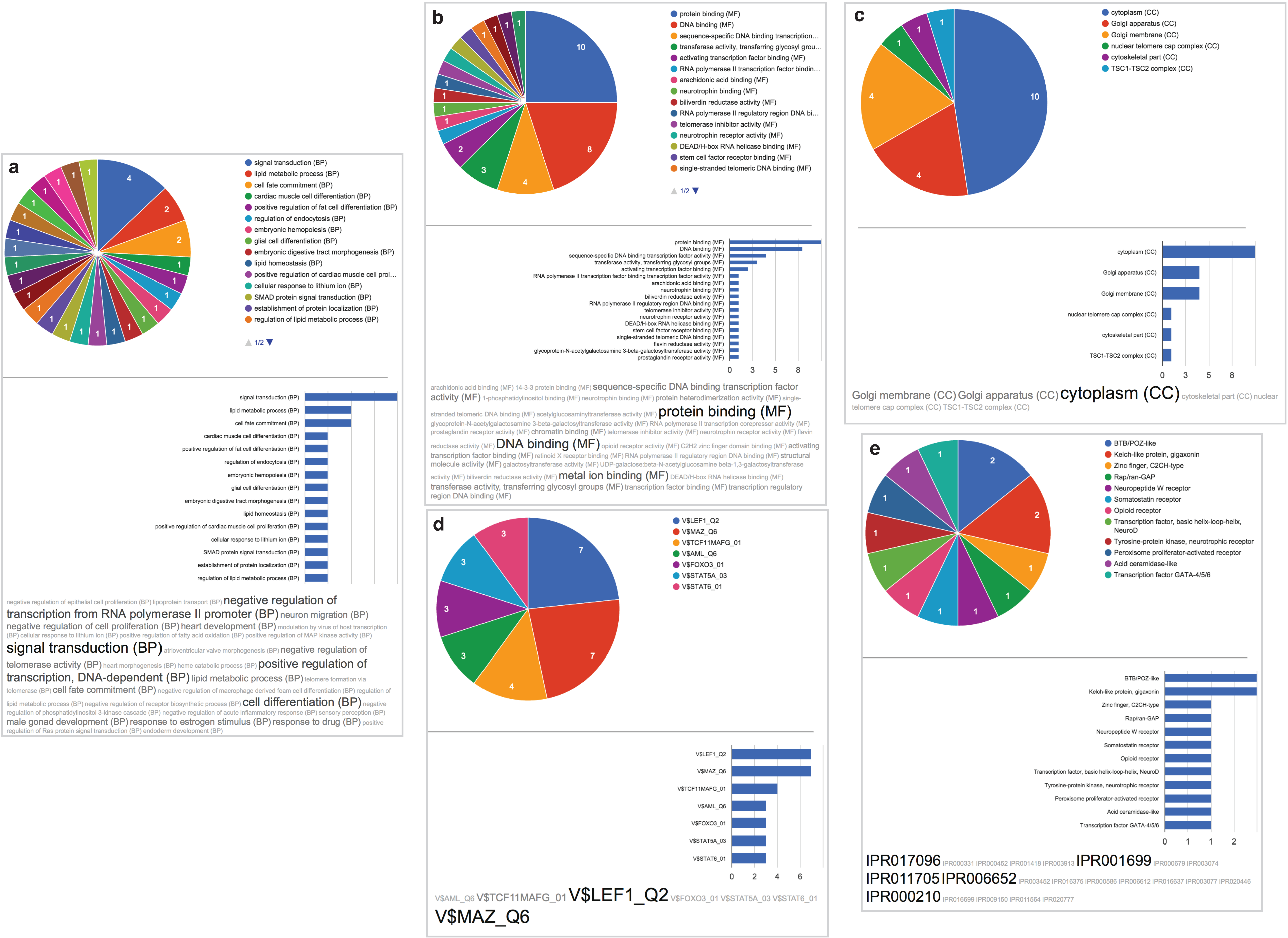

Supplementary Fig. S6: Enrichment pie charts in *B2* vs. *Ref*

Snap shots of the interactive pie charts and bar graphs obtained from Gencodis3, under following enriched annotations: (a) GO Biological processes; (b) GO Molecular function; (c) GO cellular component; (d) Transcription factors; and (e) InterPro motifs, for up-regulated DEGs identified between *B2* vs. *Ref*. Size of the slices and the length of the bars are proportional to the number of genes corresponding to the assigned annotation

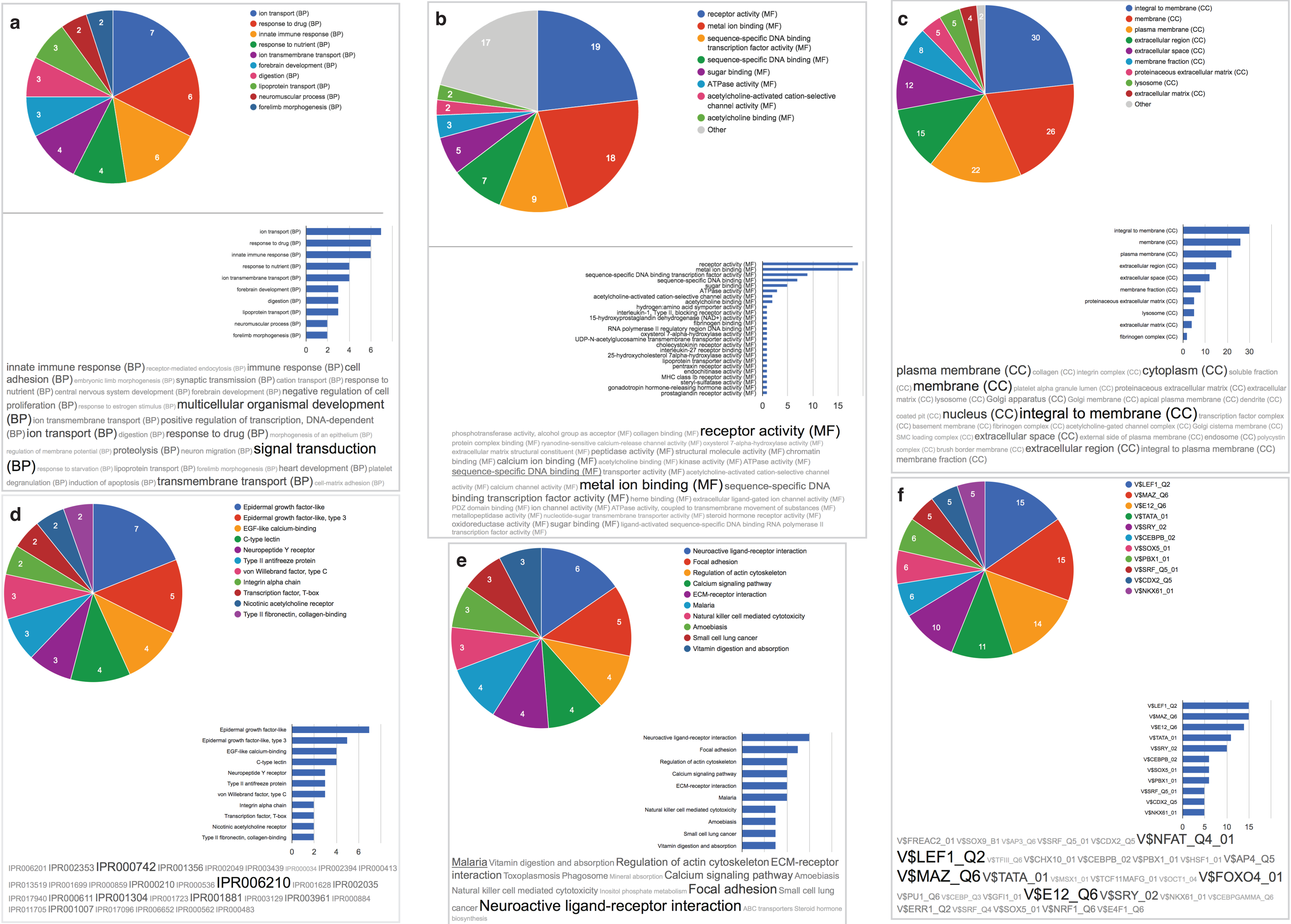

Supplementary Fig. S7: Enrichment Pie charts in *B2* vs. *A2*

Snap shots of the interactive pie charts and bar graphs obtained from Gencodis3, under following enriched annotations: (a) GO Biological processes; (b) GO Molecular function; (c) GO cellular component; (d) InterPro motifs; (e) KEGG Pathways; and (f) Transcription factors, for up-regulated DEGs identified between *B2* vs. *A2* Size of the slices and the length of the bars are proportional to the number of genes corresponding to the assigned annotation

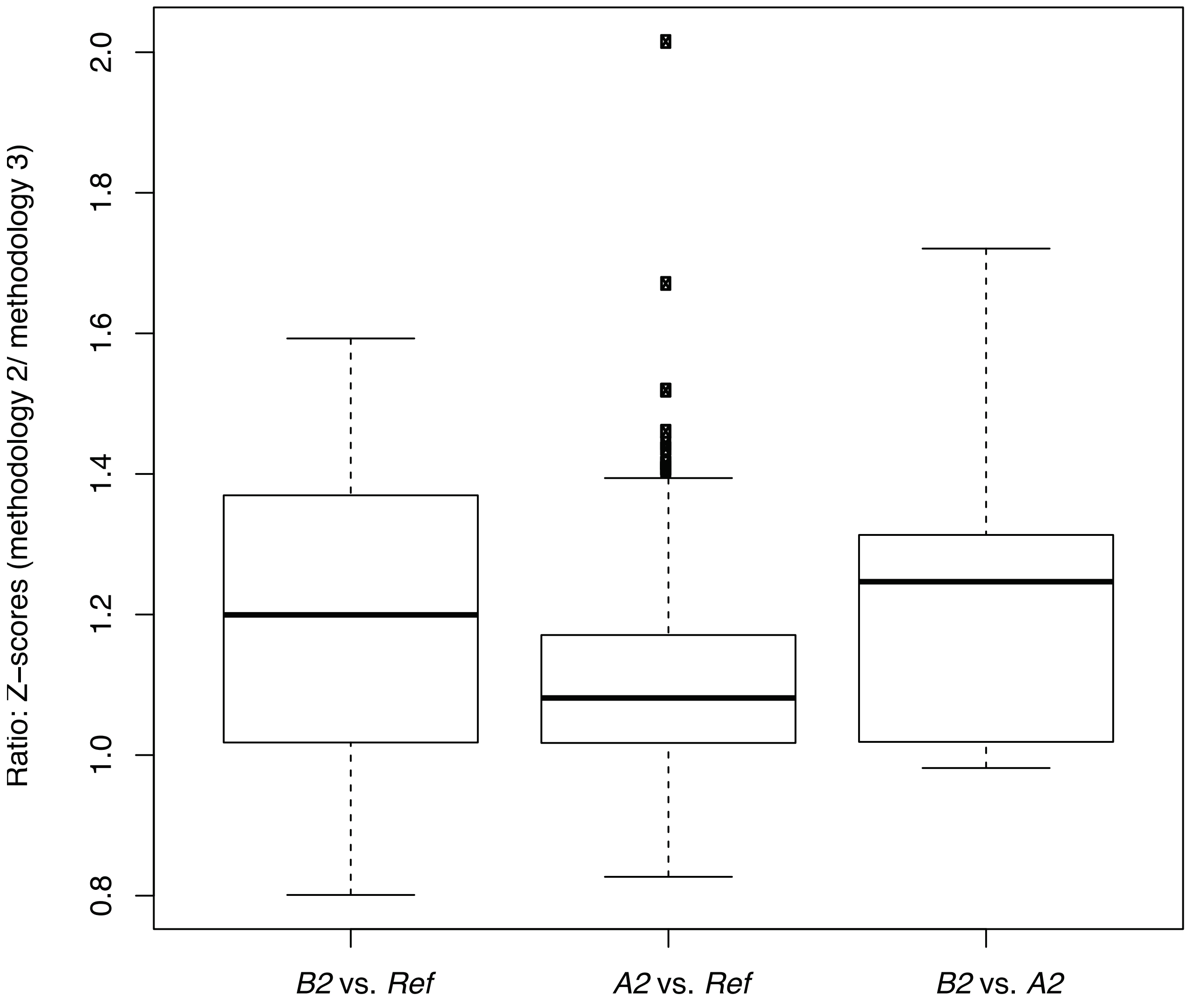

Supplementary Fig. S8: Comparison of Z-score distribution between methodology 2 and 3
For each comparison *B2* vs. *Ref*, *A2* vs. *Ref* and *B2* vs. *A2*, the ratio of Z-scores from methodology 2/ methodology 3 has been calculated for differentially expressed genes overlapping between methodology 2 and methodology 3 and plotted as boxplot to visualize the distribution of ratios. The box shows the first and third quartile (25-75%), the notches shows 95% confidence interval of median while the median is represented by bold line. For *B2* vs. *Ref*: n=57; *A2* vs. *Ref:* n=252 and *B2* vs. *A2:* n=42.
